# Intravenous iron alone resolves anemia in patients with functional iron deficiency and lymphoid malignancies undergoing chemotherapy

**DOI:** 10.1007/s12032-014-0302-3

**Published:** 2014-11-06

**Authors:** Michael Hedenus, Torbjörn Karlsson, Heinz Ludwig, Beate Rzychon, Marcel Felder, Bernard Roubert, Gunnar Birgegård

**Affiliations:** 1 Department of Internal Medicine, Sundsvall Hospital, 851 86 Sundsvall, Sweden; 2 Department of Medical Sciences, Uppsala University, Uppsala, Sweden; 3 1st Department of Internal Medicine, Center for Oncology and Hematology, Wilhelminen Hospital, Vienna, Austria; 4 Vifor Pharma, Glattbrugg, Switzerland

**Keywords:** Ferric carboxymaltose, Intravenous iron, Anemia, Lymphoid malignancies, Functional iron deficiency

## Abstract

This randomized trial evaluated ferric carboxymaltose without erythropoiesis-stimulating agents (ESA) for correction of anemia in cancer patients with functional iron deficiency. Patients on treatment for indolent lymphoid malignancies, who had anemia [hemoglobin (Hb) 8.5–10.5 g/dL] and functional iron deficiency [transferrin saturation (TSAT) ≤20 %, ferritin >30 ng/mL (women) or >40 ng/mL (men)], were randomized to ferric carboxymaltose (1,000 mg iron) or control. Primary end point was the mean change in Hb from baseline to weeks 4, 6 and 8 without transfusions or ESA. Difficulties with patient recruitment led to premature termination of the study. Seventeen patients (8 ferric carboxymaltose and 9 control) were included in the analysis. In the ferric carboxymaltose arm, mean Hb increase was significantly higher versus control at week 8 (*p* = 0.021). All ferric carboxymaltose-treated patients achieved an Hb increase >1 g/dL (control 6/9; *p* = 0.087), and mean TSAT was >20 % from week 2 onwards. No treatment-related adverse events were reported. In conclusion, ferric carboxymaltose without ESA effectively increased Hb and iron status in this small patient population.

**Electronic supplementary material:**

The online version of this article (doi:10.1007/s12032-014-0302-3) contains supplementary material, which is available to authorized users.

## Introduction

Anemia and iron deficiency are frequent complications in cancer patients, particularly in those undergoing chemotherapy [1]. Both correlate with poor performance status in cancer patients, and anemia has been shown to be associated with shorter survival [1, 2].

Inadequate iron supply is a major component in the pathogenesis of anemia in cancer patients [3].The estimated prevalence of insufficient iron availability in cancer patients ranges from 19–63 %, and functional iron deficiency (FID) is much more common than absolute iron deficiency [1, 3]. FID occurs when release of iron from internal stores is restricted (e.g., due to inflammation) or too slow to keep pace with erythropoiesis [e.g., after treatment with erythropoiesis-stimulating agents (ESA)]. It is characterized by low transferrin saturation (TSAT ≤ 20 %) in spite of adequate iron stores, while serum ferritin levels usually are elevated [3, 4].

Present management of cancer-related anemia often consists of red blood cell (RBC) transfusions or ESA treatment [5]. However, at least 30 % of anemic cancer patients do not respond to ESA treatment alone [6], and over recent years, the evidence has accumulated that RBC transfusions, as well as ESA use outside the current label and guidelines, can increase all-cause mortality [7–9]. Current guidelines therefore recommend preventing RBC transfusions and using ESAs at the lowest effective dose [10]. Randomized, controlled trials using intravenous (i.v.) iron treatment in combination with ESA showed increased hematological response, reduced RBC transfusion and ESA dose requirements, and faster correction of cancer-related anemia compared with ESA alone or with oral iron [11]. Initiation of anemia treatment with i.v. iron alone could be an interesting therapeutic option for patients with cancer-related anemia. So far, there is evidence on the benefit of i.v. iron alone in three randomized controlled studies in patients with gynecological cancers [12–14] and two observational studies [15, 16].

In this study for the first time, a distinction was made between the two basic types of iron deficiency (absolute or functional). Here, we evaluated the efficacy of i.v. ferric carboxymaltose (FCM) without the addition of ESA as treatment for chemotherapy-induced anemia in patients with lymphoid malignancies and functional iron deficiency receiving antineoplastic therapy.

## Materials and methods

This randomized, controlled, open-label, prospective trial included 11 recruiting sites across four countries (Austria, Germany, Russia, and Sweden). The study was registered (ClinicalTrials.gov Identifier: NCT01101399) and conducted in accordance with the Declaration of Helsinki and approved by Independent Ethics Committees.

Included were adult patients with lymphoid malignancies (indolent non-Hodgkin’s lymphoma, multiple myeloma, or chronic lymphocytic leukemia), anemia [hemoglobin (Hb) 8.5–10.5 g/dL], and FID [TSAT ≤ 20 % and serum ferritin >30 ng/mL (women) or >40 ng/mL (men)] who had received antineoplastic therapy for ≥8 weeks (or two cycles) prior to inclusion (see supplementary data for a comprehensive list of inclusion/exclusion criteria).

Based on a predefined, computer-generated randomization list, patients were randomized 1:1 to FCM (Ferinject^®^, Vifor Pharma, Switzerland) or no anemia treatment (controls; symptomatic management according to local institutional practice). Patients >50 kg received a single infusion of 1,000 mg iron at day 1 of the next antineoplastic therapy cycle, and patients <50 kg received two infusions of 500 mg iron each (day 1 and week 2).

Primary end point was the mean Hb change from baseline to week 8 without use of transfusions or ESA. Secondary end points included safety, Hb response (increase ≥ 1.0 g/dL) and correction (Hb ≥ 11.0 g/dL) at any week, median time to Hb response and changes in hematologic variables.

The planned sample size of 40 patients (20 per group) was calculated to detect an expected difference in Hb of 1.0 g/dL (standard deviation 1.5 g/dL) at a 1-sided alpha of 0.05 and a power of 68 %. These data were derived from a large observational study in cancer patients who had received 500–4,000 mg of FCM for the treatment of iron-restricted erythropoiesis and anemia, and achieved a ≥1.0 g/dL mean Hb increase from baseline by week 4 [15].

The primary efficacy variable was analyzed using a mixed effects model for repeated measures (MMRM). Comparisons at weeks 8, 6, and 4 were made using a hierarchical step-down procedure with the primary end point at week 8. Exact logistic regression, adjusted for baseline Hb, was used for all tests based on proportions. Time-to-event analyses were investigated using Kaplan–Meier survival plots with log-rank tests for comparisons. No adjustment was made for multiplicity.

Difficulties with patient recruitment led to premature study termination, after randomization of only half of the planned patients.

## Results

**Fig. 1:**
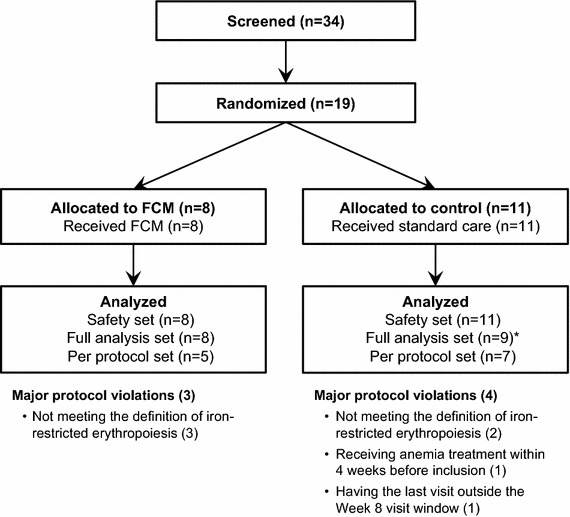
Patient flow diagram

Of 34 screened patients, 19 were randomized (safety set: 8 FCM, 11 controls) and 17 had post-baseline efficacy data available for primary endpoint analysis [full analysis set (FAS): 8 FCM, 9 controls; Fig. 1]. The per-protocol set (FAS patients with no major protocol deviations) included 12 patients (5 FCM, 7 controls). Baseline patient characteristics were comparable between the FCM and the control group (Table 1).

**Table 1:** Baseline patient characteristics (safety set)

		FCM (*n* = 8)	Controls^d^ (*n* = 11)
Male, *n* (%)		5 (62.5)	7 (63.6)
Age (years, median [range])		69.5 [41–79]	71.0 [26–88]
Weight (kg, median [range])		67.8 [59.0–103.7]	66.4 [49.0–78.0]
Baseline Hb and iron status	Hb (g/dL, median [range])	9.5 [9.0–10.5]	9.8 [8.4–10.6]
	Ferritin (ng/mL, median [range])^a^	216 [65–800]	322 [8–707]
	TSAT (%, median [range])^a^	16 [3–35]	18 [0–31]
Previous anti-anemic therapy^b^, *n* (%)	Transfusion	3 (37.5)	1 (9.1)
	ESA	0	1 (9.1)
	iron	0	1 (9.1)
Tumor type, *n* (%)	Multiple myeloma	6 (75.0)	5 (45.5)
	Chronic lymphocytic leukemia	1 (12.5)	1 (9.1)
	Non-Hodgkin’s lymphoma	1 (12.5)	5 (45.5)
Cancer therapy Mono- or combined (*n*)^c^	Antineoplastic agents	8	10
	Bendamustine	0	1
	Bortezomib	3	3
	Chlorambucil	1	2
	Cyclophosphamide	3	2
	Doxorubicin	0	1
	Fludarabine	0	1
	Melphalan	2	5
	Vincristine	1	1
	Thalidomide	1	1
	Corticosteroids for systemic use	7	6
	Dexamethasone	4	3
	Prednisone	3	4

^a^Patients with baseline TSAT >20 % and ferritin ≤30 (women) or ≤40 ng/mL (men) were excluded from the per-protocol population (3 FCM, 2 Control)

^b^>4 weeks prior to baseline

^c^As per protocol, patients had to be receiving cancer treatment

^d^Symptomatic management according to local institutional practice

In the FCM group, five patients received a single FCM administration (1,000 mg iron) and three received two FCM administrations (500 mg iron per dose).

**Fig. 2:**
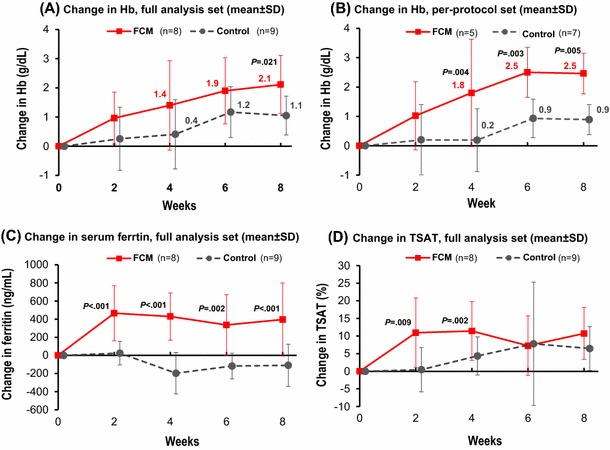
Mean change from baseline in Hb and iron status

No patient required blood transfusion or ESA treatment during the study period. In the FAS population, FCM-treated patients had a greater mean Hb increase from baseline compared with control patients at all post-baseline visits, with a statistically significant difference at week 8 (*p* = 0.021 vs. controls; Fig. 2a). Thus, the primary end point was met despite the small number of patients. Median Hb increase from baseline to week 8 was 2.1 g/dL [range 0.2–3.5 g/dL] in FCM-treated patients vs. 0.9 g/dL [range 0.3–2.2 g/dL] in the control group. In the per-protocol set, the primary endpoint analysis showed significantly higher Hb increases in the FCM versus control group from week 4 onwards (all *p* ≤ 0.005; Fig. 2b).

All (8/8) FCM-treated patients compared to 66.7 % (6/9) of control patients achieved an Hb response (*p* = 0.954). Median time to response was 2.3 [range 0.6–7.3] weeks in FCM-treated versus 4.4 [range 1.0–8.1] weeks in control patients (*p* = 0.087). Hb was corrected in 87.5 % (7/8) of FCM-treated and 55.6 % (5/9) of control patients (*p* = 0.105). In the FCM group, a median Hb of 11.8 g/dL [range 9.4–13.1 g/dL] was achieved at week 6 and remained stable until end of study (11.9 g/dL at week 8). In the control group, median Hb remained below 11.0 g/dL at all time points.

Ferritin and TSAT increased faster and to significantly higher levels in FCM-treated patients vs. controls (Fig. 2c, d). In the FCM group, a median TSAT of 28 % [range 19–38 %] was reached at week 2 and remained stable until end of study, whereas in the control group, median TSAT remained below 20 % at all time points. Changes in other tested variables (erythropoietin, hepcidin-25, and interleukin-6) were statistically not significantly different between treatment groups (supplementary Table S1).

As expected for an open-label comparison of active treatment versus controls, more treatment-emergent adverse events (TEAEs) were reported in the FCM than in the control group (FCM: 12 TEAEs in 5 patients; controls: 2 TEAEs in 1 patient; supplementary Table S2 and S3). However, none of the reported TEAEs were considered related to the study drug or led to discontinuation. No hypersensitivity reactions were observed.

## Discussion

Data from randomized controlled studies on the effect of i.v. iron as sole anemia therapy in cancer patients are still scarce, and this is the first randomized controlled trial to investigate whether i.v. iron can overcome the iron sequestration which is a consequence of the inflammatory nature of malignant disease. A single dose of FCM without concomitant ESA therapy resulted in significantly increased Hb levels which were maintained for at least 8 weeks in this population of cancer patients with lymphoid malignancies, anemia, and functional iron deficiency receiving antineoplastic therapy.

There was a large mean increase in Hb from baseline to week 8 in the FCM group (2.1 g/dL) and the primary end point was met despite the small number of evaluable patients. Using the per-protocol population, the primary endpoint analysis showed statistically significantly higher Hb increases in the FCM vs. control group from week 4 onwards. TSAT and serum ferritin increased rapidly and remained high from the first post-treatment visit onwards. No patient required a blood transfusion or an ESA treatment during the study period.

We observed a slight Hb increase in the controls at week 6–8 (Fig. 2a, b), which may be related to fluctuations in Hb levels and/or tumor regression. The latter would be in-line with the observed decrease in ferritin and concomitant TSAT increase in the controls, possibly indicating reduced inflammation and consequently increased availability of iron for erythropoiesis.

Of note, since this study was prematurely terminated due to difficulties with patient recruitment, only half of the planned patients were randomized. Thus, as a consequence, the power of the study is below the adequate power, and therefore, only limited interpretation of the data is possible. However, our results are in-line with those of a large observational study in FCM-treated anemic cancer patients (*n* = 420; 233 patients received FCM alone), which showed improvement in median Hb from 10.0 g/dL to above 11 g/dL within 5 weeks and a median Hb increase of 1.4 g/dL. This improvement was achieved without transfusions and/or ESA treatment [15]. In addition, three randomized, controlled clinical trials have shown that i.v. iron alone significantly reduced transfusion requirements in patients with gynecological cancers [12–14]. A single-arm pilot study with i.v. iron sucrose in anemic, non-iron-deficient cancer patients undergoing chemotherapy therapy without ESA use, showed a significant improvement in Hb levels compared with baseline [16]. However, in these studies no distinction was made between absolute iron deficiency (low serum ferritin) and FID, while in the present study, only patients with FID were included.

Current guidelines for cancer and chemotherapy-induced anemia recommend preventing blood transfusions and using ESAs with the lowest effective dose and in approved indications only, based on safety concerns associated with both treatments [10, 17]. In line with these recommendations, the use of i.v. iron alone in anemia therapy may reduce or even prevent the need for ESAs and RBC.

In conclusion, our study clearly indicates that i.v. iron alone corrects anemia and FID in cancer patients undergoing antineoplastic therapy. Further, appropriately powered and controlled studies are warranted to validate these results.

## Electronic supplementary material

Below is the link to the electronic supplementary material.

Supplementary material 1 (PDF 128 kb)
