# Supplementary material for: Intravenous iron alone resolves anemia in patients with functional iron deficiency and lymphoid malignancies undergoing chemotherapy
Source: Med Oncol. 2014 Nov 6;31(12):302. doi: 10.1007/s12032-014-0302-3 (PMC4221625; doi:10.1007/s12032-014-0302-3)
Supplement: Supplementary file 1 — Supplementary material 1 (PDF 128 kb) [file 12032_2014_302_MOESM1_ESM.pdf]

**Intravenous iron alone resolves anemia in patients with functional iron deficiency and lymphoid malignancies undergoing chemotherapy**

Michael Hedenus, Torbjörn Karlsson, Heinz Ludwig, Beate Rzychon, Marcel Felder, Bernard Roubert[4], and Gunnar Birgegård

Corresponding author: M. Hedenus, michael.hedenus@lvn.se

## SUPPLEMENTARY MATERIALS

## Inclusion/exclusion criteria

Included were adult patients (≥18 years old) with indolent non-Hodgkin's lymphoma, multiple myeloma or chronic lymphocytic leukemia, who had confirmed anemia (hemoglobin [Hb] 8.5-10.5g/dL) and FID (TSAT ≤20% and serum ferritin >30ng/mL [women] or >40ng/mL [men]) at the time of randomization and an assumed life expectancy of at least 6 months. Patients had to be on antineoplastic therapy and had to have been treated for ≥8 weeks (or two cycles) prior to inclusion. Women of child-bearing potential had to have had a negative urine pregnancy test at screening.

Exclusion criteria were any anemia treatment within 4 weeks prior inclusion (including red blood cell transfusion, ESA treatment, and any oral/parenteral iron supplementation), a body weight <34kg, an increase in Hb during antineoplastic therapy (>1g/dL rise between initiation of antineoplastic therapy and screening laboratory value), folate deficiency (serum folate <4.5nmol/L) and/or vitamin B12 deficiency (serum cobalamin <145pmol/L), ongoing hemolysis (serum-haptoglobin <0.2g/L), recent significant bleeding/surgery, known chronic renal failure (serum creatinine >125µmol/L), clinically relevant active inflammatory disease other than the malignant disease, clinically relevant ongoing infectious disease including known human immunodeficiency virus, serum ferritin >800ng/mL, ongoing significant neurological or psychiatric disorders including psychotic disorders or dementia, significant cardiovascular disease prior to study inclusion, known sensitivity to any of the products to be administered, elevation of liver enzymes (aspartate aminotransferase, alanine aminotransferase) over 3 times the upper normal limit range or known acute hepatic disorders. Women who were not using adequate contraceptive precautions or were evidently pregnant or breast feeding were also excluded.

**Table S1. Changes in endogenous EPO, hepcidin-25 and IL-6 levels (full analysis set)**

| | Group | Baseline [range] | Median change from baseline [range] | | | |
|---|---|---|---|---|---|---|
| | | | Week 2 | Week 4 | Week 6 | Week 8 |
| **EPO*** (IU/L) | FCM | 44 [24–314] | -4 [-105–5] | -17 [-205–(-5)] | -22 [-144–(-9)] | -23 [-148–(-8)] |
| | Control | 34 [9–176] | 5 [-24–109] | -10 [-161–1] | -19 [-68–6] | -10 [-140–16] |
| **Hepcidin-25†** (nmol/L) | FCM | 6.6 [0.5–34.6] | 3.3 [-12.1–11.4] | 0.3 [-9.4–7.7] | 0.4 [-11.8–2.6] | -0.1 [-9.4–3.4] |
| | Control | 3.9 [0–38.7] | 1.3 [-38.5–4.8] | -0.8 [-38.4–0.8] | -0.1 [-34.1–42.9] | -1.7 [-30.1–4.1] |
| **IL-6*** (pg/mL) | FCM | 9.2 [0.7–36.6] | -1.3 [-12.3–4.9] | -5.2 [-18.7–3.3] | -2.6 [-24.8–6.1] | 0.9 [-31.6–10.3] |
| | Control | 13.4 [5–300] | -2.5 [-285–51] | -4.7 [-270–0] | -2.0 [-262–21] | -3.9 [-199–11] |

* Measured by ELISA

† Measured by ultra-high-pressure liquid chromatography and a linear ion trap mass spectrometer (Bansal,S.S et al. Quantitation of hepcidin in serum using ultra-high-pressure liquid chromatography and a linear ion trap mass spectrometer. Rapid Commun Mass Spectrom. 2010;24: 1251-1259.)

Abbreviations: EPO, erythropoietin; IL-6, interleukin-6.

**Table S2. Summary of TEAEs**

| | FCM (n=8) | | Control (n=11) | |
|---|---|---|---|---|
| | n (%) | Events | n (%) | Events |
| **Any AE** | 5 (62.5%) | 12 | 1 (9.1%) | 2 |
| **Any treatment-related AE** | 0 | 0 | 0 | 0 |
| **Any severe AE** | 1 (12.5%) | 1* | 0 | 0 |
| **Any AE with outcome of death** | 1 (12.5%) | 1* | 0 | 0 |
| **Any serious AE** | 1 (12.5%) | 2* | 0 | 0 |
| **Any AE leading to discontinuation** | 0 | 0 | 0 | 0 |

* Post-study event: unrelated death after injury; in the same patient: moderate atrial fibrillation at Day 49.

Abbreviations: TEAEs, treatment-emergent adverse events; AE, adverse event; FCM, ferric carboxymaltose.

**Table S3. TEAEs* occurring in ≥2 patients by system organ class**

| | FCM (n=8) | Control (n=11) |
|---|---|---|
| | n (%) | n (%) |
| **Infections and infestations** | 3 (37.5%) | 1 (9.1%) |
| Herpes Zoster | 2 (25.0%) | 0 |
| URTI | 1 (12.5%) | 0 |
| Pneumonia fungal | 0 | 1 (9.1%) |
| **Gastrointestinal disorders** | 2 (25%) | 0 |
| Diarrhea | 1 (12.5%) | 0 |
| Nausea | 1 (12.5%) | 0 |
| **Musculoskeletal and connective tissue disorders** | 2 (25.0%) | 0 |
| Back pain | 1 (12.5%) | 0 |
| Bone pain | 1 (12.5%) | 0 |

*None was considered related to the study drug.

Abbreviations: TEAEs, treatment-emergent adverse events; URTI, upper respiratory tract infection; FCM, ferric carboxymaltose.
